# Aqueous extract of *Amydrium sinense (Engl.) H. Li* alleviates hepatic fibrosis by suppressing hepatic stellate cell activation through inhibiting Stat3 signaling

**DOI:** 10.3389/fphar.2023.1101703

**Published:** 2023-06-13

**Authors:** Jingyan Li, Bingmin Wu, Lishan Zeng, Ying Lin, Qiuhe Chen, Haixia Wang, Lin An, Jiajun Zhang, Siyan Chen, Junying Huang, Ruoting Zhan, Guifang Zhang

**Affiliations:** ^1^ Guangdong Key Laboratory for Translational Cancer Research of Chinese Medicine, Joint Laboratory for Translational Cancer Research of Chinese Medicine of the Ministry of Education of the People’s Republic of China, International Institute for Translational Chinese Medicine, School of Pharmaceutical Sciences, Guangzhou University of Chinese Medicine, Guangzhou, Guangdong, China; ^2^ College of Life Sciences, Guangzhou University, Guangzhou, Guangdong, China; ^3^ Key Laboratory of Chinese Medicinal Resource from Lingnan, Ministry of Education, Guangzhou University of Chinese Medicine, Guangzhou, China

**Keywords:** *Amydrium sinense*, hepatic fibrosis, carbon tetrachloride, α-SMA, Stat3

## Abstract

**Background:** The present study aimed to investigate the protective effect of the water extract of *Amydrium sinense* (Engl.) H. Li (ASWE) against hepatic fibrosis (HF) and clarify the underlying mechanism.

**Methods:** The chemical components of ASWE were analysed by a Q-Orbitrap high-resolution mass spectrometer. In our study, an *in vivo* hepatic fibrosis mouse model was established via an intraperitoneal injection of olive oil containing 20% CCl_4_. *In vitro* experiments were conducted using a hepatic stellate cell line (HSC-T6) and RAW 264.7 cell line. A CCK-8 assay was performed to assess the cell viability of HSC-T6 and RAW264.7 cells treated with ASWE. Immunofluorescence staining was used to examine the intracellular localization of signal transducer and activator of transcription 3 (Stat3). Stat3 was overexpressed to analyse the role of Stat3 in the effect of ASWE on HF.

**Results:** Gene Ontology (GO) and Kyoto Encyclopedia of Genes and Genomes (KEGG) analyses showed that candidate targets of ASWE, associated with protective effects against hepatic fibrosis, were related to inflammation response. ASWE ameliorated CCl_4_-induced liver pathological damage and reduced the liver index and alanine transaminase (ALT) and aspartate transaminase (AST) levels. ASWE also decreased the serum levels of collagen Ⅰ (Col Ⅰ) and hydroxyproline (Hyp) in CCl_4_-treated mice. In addition, the expression of fibrosis markers, including α-SMA protein and *Acta2*, *Col1a1*, and *Col3a1* mRNA, was downregulated by ASWE treatment *in vivo*. The expression of these fibrosis markers was also decreased by treatment with ASWE in HSC-T6 cells. Moreover, ASWE decreased the expression of inflammatory markers, including the *Tnf-α*, *Il6* and *Il1β*, in RAW264.7 cells. ASWE decreased the phosphorylation of Stat3 and total Stat3 expression and reduced the mRNA expression of the *Stat3* gene *in vivo* and *in vitro*. ASWE also inhibited the nuclear shuttling of Stat3. Overexpression of Stat3 weakened the therapeutic effect of ASWE and accelerated the progression of HF.

**Conclusion:** The results show that ASWE protects against CCl_4_-induced liver injury by suppressing fibrosis, inflammation, HSC activation and the Stat3 signaling pathway, which might lead to a new approach for preventing HF.

## 1 Introduction

Hepatic fibrosis (HF) is a challenging clinical disease and a reversible wound-healing response during liver injury repair. HF is observed in patients with chronic viral hepatitis, nonalcoholic fatty liver disease, alcoholic liver disease, obesity and cholestatic and autoimmune liver diseases (Mederacke et al., 2013; Zheng et al., 2013). Without effective treatment, the continued development of HF results in the development of cirrhosis and hepatoma, leading to increased mortality due to liver disease worldwide (Hernandez-Gea and Friedman, 2011; Roeb, 2018). It is generally recognized that hepatic stellate cells (HSCs) are critical in the occurrence and development of HF. In response to various complex adverse factors, quiescent HSCs are activated and transdifferentiate into myofibroblast-like cells, leading to the excessive deposition of extracellular matrix (ECM), the formation of fibrotic nodules, and ultimately the acceleration of HF. Numerous experimental and clinical studies have also confirmed that inhibiting HSC activation is a potentially effective strategy for the treatment of HF (Lee et al., 2015; Tsuchida and Friedman, 2017).

Inflammation, which is one of the most prominent characteristic features of HF, is thought to accelerate the further development of HF due to the critical role of inflammation in the underlying pathogenesis of HF (Seki and Schwabe, 2015). Recent studies have noted that genes that regulate the inflammatory response to injury determine the fibrotic response to injury. For example, tumor necrosis factor-α (Tnf-α), interleukin 1β (Il1β), and interleukin 6 (Il6) may trigger the accumulation of associated cells (e.g., neutrophils) that drive the early stages of disease progression and maintain ongoing inflammation in the liver (Duffield et al., 2005; Mitchell et al., 2009). Typically, HF patients have elevated serum levels of inflammatory cytokines (Liaskou et al., 2013). HSCs are the main source of ECM, and can be activated by inflammation or mechanical stimulation, thus promoting the development of HF and the reconstruction of the intrahepatic structure (Kisseleva and Brenner, 2007; Lu et al., 2015). HSCs are highly sensitive to proinflammatory cytokines, leading to the activation of proinflammatory signaling pathways such as signal transducer and activator of transcription 3 (Stat3) and the subsequent production of chemokines and cytokines (Seki and Schwabe, 2015). Stat3 has been highlighted as a regulator of many biological processes, including cell survival, apoptosis, inflammation and angiogenesis (Zou et al., 2020; Xiao et al., 2022). Stat3 is essential for transducing fibrotic signaling in HF. Several studies have demonstrated that sustained activation of Stat3 promotes inflammation, leading to various pathological manifestations of HF, such as increased the expression of the fibrosis markers alpha smooth muscle actin (α-SMA), collagen 1 (Col Ⅰ) and collagen 3 (Col Ⅲ) (Deng et al., 2013; Xiang et al., 2018). Blockade of the Stat3 signaling pathway inhibits the morphological transdifferentiation of HSCs and reduces the mRNA expression of profibrotic genes (Wang et al., 2018).

To date, the pathogenesis of HF has been widely examined. However, precise and efficient drugs for treating HF have not been successfully identified (Schuppan et al., 2018). The ethnic medicine *Amydrium sinense (Engl.) H. Li* (AS) is the dried whole herb of the genus *Amydrium* in the family *Araceae*. This Chinese vine has long been used to treat diseases. According to the literature and related books, AS is mainly used in folk medicine to treat common diseases related to acute and chronic tissue inflammation, such as rheumatism, angina pectoris, fractures, bruises, and sprains. In our preliminary studies, we found that the water extract of AS (ASWE) could markedly inhibit the activation of HSCs. Therefore, we hypothesized that ASWE might protect against HF. Therefore, the present study was designed to analyse the chemical composition of ASWE, investigate the antihepatic fibrosis effect of ASWE in combination with *in vitro* and *in vivo* experiments, and clarify the underlying mechanism.

## 2 Materials and methods

### 2.1 Chemicals and antibodies

Carbon tetrachloride, methanol, acetonitrile, chloroform, isopropanol, anhydrous ethanol, and formic acid were purchased from Macklin (Shanghai, China). Transforming growth factor-β1 (TGF-β1) and lipopolysaccharide (LPS) were purchased from Aladdin (Shanghai, China). Fetal bovine serum (FBS), penicillin/streptomycin solution and Trizol reagent were obtained from Thermo Fisher Scientific. Dimethyl sulfoxide was purchased from Sigma-Aldrich Corporation (St. Louis, MO, United States). Trypsin was obtained from Invitrogen (Carlsbad, CA, United States). The enhanced BCA protein assay kit (P0010), Alexa Fluor 488-conjugated anti-mouse IgG (H + L) secondary antibody, and DAPI staining solution were purchased from Beyotime Biotechnology (Shanghai, China).

Antibodies against α-SMA (14395-1-AP), Stat3 (10253-2-AP), and GAPDH (6,004-1-lg) were obtained from Proteintech (Chicago, United States). Antibody against phospho-Stat3 (p-Stat3) (Tyr705) was purchased from Cell Signaling Technology (Boston, MA, United States). Goat anti-rabbit IgG (H + L) secondary antibody (BS13278), and Goat anti-mouse IgG (H + L) secondary antibody (BS12478) were procured from Bioworld Technology (St. Paul, MN, United States).

### 2.2 Herb collection and extraction

The herbs used in the experiment were collected from Yangshan County, Qingyuan City, Guangdong Province, and were identified as the whole plant of *Amydrium sinense (Engl.) H. Li* by Zhang Guifang, Associate Professor, Guangzhou University of Traditional Chinese Medicine. The dried whole herb of *Amydrium sinense (Engl.) H. Li* was soaked for 1 h in advance and then decocted thrice with 20-fold volumes of distilled water for 1 h each. The drug solutions were combined and filtered. Afterwards, those drug solutions were concentrated under reduced pressure and freeze-dried to obtain the water extract of *Amydrium sinense (Engl.) H. Li* (ASWE).

### 2.3 Analysis of ASWE by Q-Orbitrap high-resolution mass

In this study, the chemical components of ASWE were analyzed by the Q-Orbitrap high-resolution mass spectrometer, which was used for the rapid identification of complex components in herbal medicines. The system was equipped with an ESI source and operated in the Full MS scan/dd-MS2 (Top N) scan mode to accurately determine the mass number of the samples and the acquisition of fragment ions. The detection instruments included Thermo Scientific™, Ultimate™3000RS, Thermo Scientific™, Q Exactive™ and RP-C18 column (150 mm × 2.1 mm, 1.8 µm). Mass spectrometry conditions were as follows: scan range, 150–2,000 m/z; aux gas heater temperature, 350°C; capillary temperature, 300°C; spray voltage, 3.8 kV; and sheath gas pressure, 40 Arb. High purity nitrogen gas (purity ≥99:999%) was used as both aux gas and sheath gas. High purity argon gas (purity ≥99:999%) was used as the collision gas. Full-mass and dd-MS2 data in positive and negative modes were obtained at 70,000 and 17,500 FWHM (full width, half maximum), respectively. Chromatography conditions were as follows: column temperature, 35°C; water phase (A), 0.1% aqueous solution of formic acid; organic phase (B), acetonitrile solution containing 0.1% formic acid. The gradient elution sequence [A:B (v/v) at time (Villesen et al.)] was set as follows: (98:2) at 0 min; (98:2) at 1 min; (80:20) at 5 min; (50:50) at 10 min; (20:80) at 15 min; (5:95) at 20 min; (5:95) at 25 min; (98:2) at 26 min; and (98:2) at 30 min. The injected sample volume was 5.00 µL, and the sample flow rate was 0.30 mL/min. All data were acquired and processed using the CD2.1 software (Thermo Fisher), and then retrieved and compared in the mzCloud, mzVault, and ChemSpider databases.

### 2.4 Prediction of targets for ASWE chemicals and identification of HF-related targets

Potential targets of main chemical compounds of ASWE were obtained from the Encyclopedia of Traditional Chinese Medicine (ETCM) Database (http://www.tcmip.cn/ETCM/index.php/Home/Index/) (Xu et al., 2019). The HF-related targets were obtained from the following databases: DisGeNET (https://www.disgenet.org/) (Piñero et al., 2020), GeneCards (https://www.genecards.org/) (Stelzer et al., 2016), Comparative Toxicogenomics Database (CTD) (https://ctdbase.org/) (Zhao et al., 2022b)). We used “hepatic fibrosis” as the search term, and the organism was restricted to *Homo sapiens*.

### 2.5 Construction of network and functional enrichment analysis

After the screening and mapping of the active compounds and active targets for ASWE were completed, Cytoscape 3.7.1. was used to construct a protein-protein interaction (PPI) network of ASWE potential targets together with HF-related targets (Martin et al., 2010). To explore the biological processes of core targets, Gene Ontology (GO) biological function and KEGG pathway enrichment analyses were performed with the online tool DAVID Bioinformatics Resources 6.8 (Chen et al., 2017). The screening criteria were set as *p* < 0.05, and the species was limited to *H. sapiens*.

### 2.6 Experimental animals and treatments

Male C57BL/6J mice (age, 8 weeks), body weight 20 ± 2 g, were procured from the Experimental Animal Center of Guangzhou University of Chinese Medicine [SCXK (Guangdong) 2019–0202]. The experimental procedures and animal care were approved by the Laboratory Animal Ethics Committee of Guangzhou University of Chinese Medicine (No. ZYD-2020–135). Mice were fed and watered *ad libitum* and maintained in a suitable environment (22°C–24°C, 45%–50% relative humidity) with a 12 h light/dark cycle. After 1 week of acclimatization, mice were randomly divided into 5 groups (*n* = 6): the control group, the CCl_4_ group, the ASWE drug-treatment groups (40, 80, 160 mg/kg/day respectively). In addition to the control group, hepatic fibrosis was induced in mice via intraperitoneally injecting (i.p.) of 20% CCl_4_-olive oil (1:4 v/v, 5 mL/kg) twice a week for 4 weeks (Liu et al., 2021a; Song et al., 2023). The control group was given the same dose of olive oil. At the beginning of modeling, all treatment groups received an intragastric administration of ASWE (dissolved in PBS) at the doses of 40, 80, and 160 mg/kg once each day for 4 weeks. On the same day, mice in the control and CCl4 groups received equal volumes of PBS using a gastric gavage. At the end of treatment, blood samples were collected from orbital sinus by rapidly removing the eyeball after the mice were anesthetized with 0.2% sodium pentobarbital (0.2 mg/kg, i.p.). Approximately 1 mL blood was collected in an EP tube for each mouse. Following blood sample collection, the mice were sacrificed by cervical dislocation. Liver tissues were obtained by a midline laparotomy.

### 2.7 Calculation of the live index

The liver tissues were washed with pre-cooled saline and blotted with filter paper. The liver weights were weighed and recorded. The liver index was expressed as (liver weight, mg)/(body weight, g) × 100%.

### 2.8 Liver histology

The morphology of liver lobes was photographed for retention. Liver tissues were fixed in 4% paraformaldehyde (Solarbio, China), sequentially dehydrated and then embedded in paraffin. The paraffin-embedded tissue samples were then sectioned into 5 μm slices and finally routinely stained with hematoxylin and eosin (H&E) and Masson’s trichrome (Solarbio, China). The images were captured under a light microscope (E100, Nikon Corporation).

### 2.9 Immunohistochemistry assay

The paraffin-embedded liver tissues were subjected to immunohistochemical staining with Stat3 antibody. Then, microscopic areas in all liver sections were randomly selected for examination, and photographed in a blinded manner using a section digital scanner (E100, Nikon Corporation).

### 2.10 Serum biochemical assay

Briefly, blood samples were kept at room temperature for 2 h and then centrifuged at 4°C (3,500 rpm, 10 min). After that, mice serum samples were collected for analysis. The serum levels of alanine transaminase (ALT) and aspartate aminotransferase (AST), which are normally used to assess liver function (Li et al., 2022b), were determined according to the instructions of the ALT (C009-2–1) and AST (C010-2–1) kits (Nanjing Jiancheng Institute of Biological Engineering, Nanjing, China). To observe collagen deposition in the liver tissues, the level of hydroxyproline (Hyp) in serum was determined using hydroxyproline assay kit. The level of Col Ⅰ in serum was detected by a standard sandwich ELISA method (E-EL-M0325c, Elabscience, Wuhan, China).

### 2.11 Cell culture and treatments

The leukemia cells in mouse macrophage (RAW264.7) and the rat hepatic stellate cells (HSC-T6) were purchased from the Cell Bank of Academy of Sciences (Shanghai, China). The cells were cultured in Dulbecco’s modified Eagle’s medium (DMEM) containing 10% FBS and penicillin/streptomycin (1:100) at 37°C in a humidified atmosphere containing 5% CO_2_.

The cells were inculated on 6-well culture plates in a serum-free conditioned medium for 12 h. Then HSC-T6 cells were stimulated with 10 ng/mL TGF-β1 for 1 h, and then ASWE (0.25 mg/mL, 0.5 mg/mL, 1.0 mg/mL) was dissolved in the DMEM supernatant containing TGF-β1 for 24 h (Dewidar et al., 2019; Zhao et al., 2022a). RAW264.7 cells were treated with 1 μg/mL LPS for 1 h. Afterwards, the cells were cultured in the DMEM supernatant containing ASWE (0.25 mg/mL, 0.5 mg/mL, 1.0 mg/mL) and LPS for 24 h (Lee et al., 2019; Zhou et al., 2021). Thereafter, total protein and RNA were extracted for subsequent experiments.

### 2.12 Cell viability assay

Cell viability was tested by a Cell Counting Kit-8 (CCK-8) assay (Dojindo Laboratories, Kyushu island, Japan). Cells, seeded in 96-well plates at equal densities for 24 h, were treated with ASWE at the concentration ranging from 0.05 to 2.50 mg/mL for 24 h. Then, CCK-8 solution (10 μL) was added to each well and incubated for 2 h at 37°C. Afterwards, the absorbance value at 450 nm was measured with a microplate reader (Thermo Varioskan LUX, MA, United States).

### 2.13 Immunofluorescent assay

To observe Stat3 nucleus shuttling, cultured cells were fixed with 4% paraformaldehyde for 10 min. Afterwards, the cells were permeabilized with 0.1% Triton X-100 for 10 min, followed by incubation with 10% goat serum for blocking the non-specific staining and incubation with Stat3 antibody overnight at 4°C. Thereafter, Alexa Fluor 488-conjugated anti-mouse IgG (H + L) secondary antibody was used to incubate in the dark for 1 h at room temperature. The nuclei were stained with DAPI solution for 10 min in the dark at room temperature. Finally, the cells were examined with a confocal microscope (LSM 710, Carl Zeiss, Germany).

### 2.14 Plasmid transfection

For overexpression studies, the Stat3 plasmid was constructed with pcDNA3.1. The plasmid was confirmed by DNA sequencing, which was performed at Sangon Biotech Co. Ltd (Shanghai, China). HSC-T6 and RAW264.7 cells were transiently transfected with the Stat3 plasmid or empty vector using Lipofectamine 2,000 reagent (Invitrogen, Carlsbad, CA, United States) according to the manufacturer’s instructions and then incubated for 48 h before harvesting.

### 2.15 Western blot analysis

Radioimmunoprecipitation assay (RIPA) buffer (Beyotime, Nantong, Jiangsu, China) supplemented with 1% protease inhibitor cocktail (Beyotime, Nantong, Jiangsu, China) was used for protein extraction from tissues and cells. The concentrations of total protein samples were determined based on the instructions of bicinchoninic acid (BCA) protein assay kit (Thermo Fisher Scientific, MA, United States). The procedure for Western blot was in the light of our previously described procedure (Li et al., 2019; Li et al., 2022a). Image-Pro Plus 6.0 software (Rockville, MD, United States) was utilized to calculate the intensity of immunoreactive bands in different lanes. The results were expressed as density values normalized to GAPDH.

### 2.16 Total RNA extraction and quantitative real-time PCR

Total RNA was extracted from the liver tissues or cells with Trizol reagent (Accurate Biotechnology, Human, China) in accordance with specific instructions. RNA concentrations and purity were assessed by the measurement of optical density at 260 and 280 nm. The mRNA levels of the target genes were determined using the SYBR Green Quantitative PCR kit (TOYOBO, Japan) as previously described (Li et al., 2016). The semi-quantitative RT-qPCR data of every target gene were expressed as 2^−ΔΔCT^ relative expression compared with endogenous GAPDH. Results were presented as fold change to control group. The primers used in the real-time PCR analysis were designed by Sangon Biotech Co (Shanghai, China). Primer sequences are listed in Table 1.

**TABLE 1:** Primer sequences used for quantitative PCR.

Species	Primers	Sequences
Mus	*Gapdh*-forward	GCC​TCG​TCC​CGT​AGA​CAA​AA
	*Gapdh*-reverse	TAC​GGC​CAA​ATC​CGT​TCA​CA
	*Acta2*-forward	GAA​GCT​CGT​TAT​AGA​AAG​AGT​GG
	*Acta2*-reverse	TCA​GGG​AGT​AAT​GGT​TGG​AAT
	*Col1a1*-forward	TTC​TCC​TGG​CAA​AGA​CGG​AC
	*Col1a1*-reverse	CGG​CCA​CCA​TCT​TGA​GAC​TT
	*Col3a1*-forward	ACG​TAA​GCA​CTG​GTG​GAC​AG
	*Col3a1*-reverse	CAG​GAG​GGC​CAT​AGC​TGA​AC
	*Tnf-α*-forward	ATG​GCC​TCC​CTC​TCA​TCA​GT
	*Tnf-α*-reverse	TTT​GCT​ACG​ACG​TGG​GCT​AC
	*Il1β*-forward	TGC​CAC​CTT​TTG​ACA​GTG​ATG
	*Il1β*-reverse	TGA​TGT​GCT​GCT​GCG​AGA​TT
	*Il6*-forward	GТССТТССТАССССААТТССА
	*Il6*-reverse	CGC​ACT​AGG​TTT​GCC​GAG​TA
	*Stat3*-forward	TGT​CAG​ATC​ACA​TGG​GCT​AAA​T
	*Stat3*-reverse	GGT​CGA​TGA​TAT​TGT​CTA​GCC​A
Rat	*Gapdh*-Forward	AGT​GCC​AGC​CTC​GTC​TCA​TA
	*Gapdh*-Reverse	GAT​GGT​GAT​GGG​TTT​CCC​GT
	*Acta2*-forward	CAT​CCG​ACC​TTG​CTA​ACG​GA
	*Acta2*-reverse	GTC​CAG​AGC​GAC​ATA​GCA​CA
	*Col1a1*-forward	GTGCGATGGCGTGCTATG
	*Col1a1*-reverse	ACT​TCT​GCG​TCT​GGT​GAT​ACA
	*Col3a1*-forward	AGA​TGC​TGG​TGC​TGA​GAA​GAA​AC
	*Col3a1*-reverse	GCT​GGA​AAG​AAG​TCT​GAG​GAA​GG

### 2.17 Statistical analysis

Data were expressed as the mean ± standard deviation (SD) from at least three independent experiments. Statistical analysis was performed using GraphPad Prism version 7.0 (San Diego, CA, United States). Statistical analyses between two groups were performed by Student’s t-test and multiple groups were performed by one-way analysis of variance (ANOVA). In all cases, a value of *p* < 0.05 was regarded to be statistically significant.

## 3 Results

### 3.1 Components in ASWE

**FIGURE 1:**
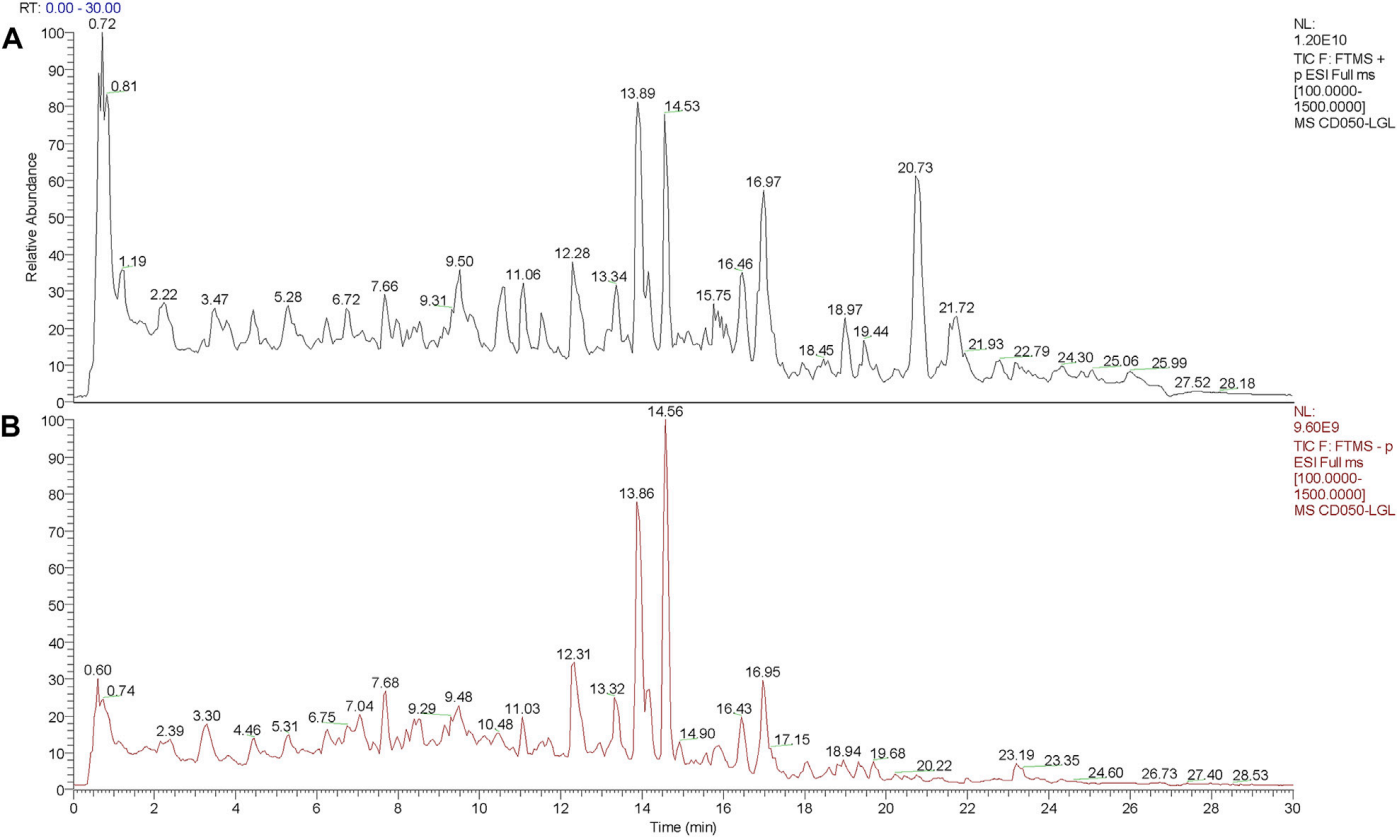
Components in ASWE. **(A)** Positive mode. **(B)** Negative mode.

Generally, the composition of aqueous extract of herbs is complex, therefore, we used the Q-Orbitrap method, which has a series of advantages (Feng et al., 2021) for the detection of main chemical components in ASWE. The total ion chromatogram of ASWE was obtained as shown in Figure 1, while a total of 45 compounds were successfully characterized as shown in Table 2, including syringic acid, neochlorogenic acid, 4-(2-Hydroxyethyl)-2-methoxyphenyl *ß*-D-glucopyranoside, coniferin, catechin, asperulosidic acid, chlorogenic acid, fraxin, fraxetin, 2-anisic acid, ageratriol, ferulic acid, suberic acid, 1,2,3,4-tetramethyl-1,3-cyclopentadiene, naringeninchalcone and other active ingredients.

**TABLE 2:** Identification of the chemical constituents obtained for ASWE.

NO.	t_R_/min	Formula	m/z	Area	Identification
1	3.15	C_9_H_10_O_5_	198.05	5,736,783	Syringic acid
2	3.25	C_16_H_18_O_9_	354.09	427,680	Neochlorogenic acid
3	3.50	C_15_H_22_O_8_	347.16	5,965,580	4-(2-Hydroxyethyl)-2-methoxyphenyl *ß*-D-glucopyranoside
4	4.72	C_16_H_22_O_8_	359.16	3,845,334	Coniferin
5	4.78	C_15_H_14_O_6_	290.08	147,186	Catechin
6	4.97	C_18_H_24_O_12_	449.15	12,057,938	Asperulosidic acid
7	5.16	C_16_H_18_O_9_	354.09	206,555	Chlorogenic acid
8	5.91	C_16_H_18_O_10_	370.09	17,855,131	Fraxin
9	5.91	C_10_H_8_O_5_	208.04	329,023	Fraxetin
10	7.06	C_8_H_8_O_3_	134.04	314,471	2-Anisic acid
11	7.10	C_15_H_24_O_3_	234.16	1,446,924	Ageratriol
12	7.89	C_10_H_10_O_4_	194.06	2,110,227	Ferulic acid
13	7.97	C_8_H_14_O_4_	174.09	1,482,813	Suberic acid
14	8.19	C_9_H_14_	122.11	2,598,184	1,2,3,4-Tetramethyl-1,3-cyclopentadiene
15	8.29	C_15_H_12_O_5_	272.07	40,728	Naringeninchalcone
16	8.56	C_26_H_34_O_11_	539.24	3,498,548	Lariciresinol 4-O-glucoside
17	8.84	C_10_H_18_O	136.13	10,407,830	Eucalyptol
18	9.39	C_21_H_20_O_12_	464.10	405,504	Quercetin-3β-D-glucoside
19	9.68	C_15_H_10_O_7_	302.04	61,506	Quercetin
20	10.00	C_9_H_16_O_4_	188.10	17,678,287	Azelaic acid
21	10.14	C_17_H_17_NO_3_	283.12	1,611,911	(2E)-3-(4-Hydroxyphenyl)-N-[2-(4-hydroxyphenyl)ethyl]acrylamide
22	10.15	C_11_H_12_O_5_	206.06	2,707,861	Sinapinic acid
23	11.08	C_21_H_20_O_10_	432.11	349,693	Afzelin
24	11.08	C_15_H_10_O_6_	286.05	137,921	Kaempferol
25	11.45	C_15_H_22_O	218.17	4,531,989	Nootkatone
26	11.53	C_18_H_28_O_3_	292.20	5,454,425	8-{3-Oxo-2-[(2E)-2-penten-1-yl]-1-cyclopenten-1-yl}octanoic acid
27	12.07	C_18_H_28_O_3_	292.20	11,601,814	9S,13R-12-Oxophytodienoic acid
28	12.88	C_15_H_24_O	220.18	16,534,693	(−)-Caryophyllene oxide
29	13.02	C_24_H_30_O_8_	446.19	6,336,847	1,4-Bis(3,4,5-trimethoxyphenyl)-hexahydrofuro[3,4-c]furan
30	13.20	C_13_H_24_N_2_O	224.19	1,205,424	N,N′-Dicyclohexylurea
31	13.36	C_18_H_32_O_5_	328.22	301,864,820	Corchorifatty acid F
32	13.97	C_18_H_34_O_4_	314.25	1,439,020	(+/−)12(13)-DiHOME
33	15.52	C_18_H_30_O_3_	294.22	65,271,268	9-Oxo-ODE
34	16.29	C_12_H_26_O_4_S	266.16	6,561,369	Dodecyl sulfate
35	16.43	C_18_H_39_O_7_P	398.24	1,004,724	Tris(2-butoxyethyl) phosphate
36	16.81	C_16_H_30_O_2_	254.22	1,358,160	Palmitoleic acid
37	16.99	C_18_H_30_O_2_	278.22	607,385,742	α-Eleostearic acid
38	17.96	C_21_H_38_O_4_	354.28	1,211,259	1-Linoleoyl glycerol
39	18.00	C_30_H_48_O_4_	489.38	182,542	Maslinic acid
40	18.52	C_10_H_10_O_3_	160.05	1,083,312	4-Methoxycinnamic acid
41	19.29	C_20_H_39_NO_2_	325.30	6,981,250	Oleoyl ethanolamide
42	19.38	C_30_H_48_O_3_	438.35	1,528,445	Oleanolic acid
43	20.63	C_19_H_39_NO	297.30	1,238,856	Tridemorph
44	22.50	C_22_H_45_NO	339.35	2,599,655	Docosanamide
45	22.74	C_10_H_12_O_2_	164.08	353,952	4-Phenylbutyric acid

### 3.2 Compound-target network analysis and functional analysis of ASWE targets for treating HF

A total of 250 putative targets of ASWE were predicted by the, ETCM database. Based on the DisGeNET, GeneCards and CTD Databases, 998 HF-related genes were collected. Screening showed that of the 250 ASWE targets, 44 were also known therapeutic targets for treating HF (Supplementary Figure S1A). To reflect the relationship between targets and compounds, we have used Cytoscape 3.7.1 to map out the compound-target relation network diagram. As shown in Supplementary Figure S1B, the compound-target diagram consisted of 52 nodes (8 core active compound nodes and 44 active target nodes). Then, to identify the biological function of the core targets, GO and KEGG enrichment analyses of the 44 core targets were performed using the DAVID online tool. As shown in Supplementary Figure S1C, 8 vital biological processes were obtained by mapping targets. The majority of these targets were closely related to the mechanism of HF. The KEGG enrichment analysis showed that the targets were notably related to the non-alcoholic fatty liver disease, alcoholic liver disease, hepatitis C, hepatitis B (Supplementary Figure S1D). These analytical results suggested that inflammatory response might be involved in the anti-antifibrotic process of ASWE against HF.

### 3.3 ASWE alleviated hepatic injury in CCl4-induced mice

**FIGURE 2:**
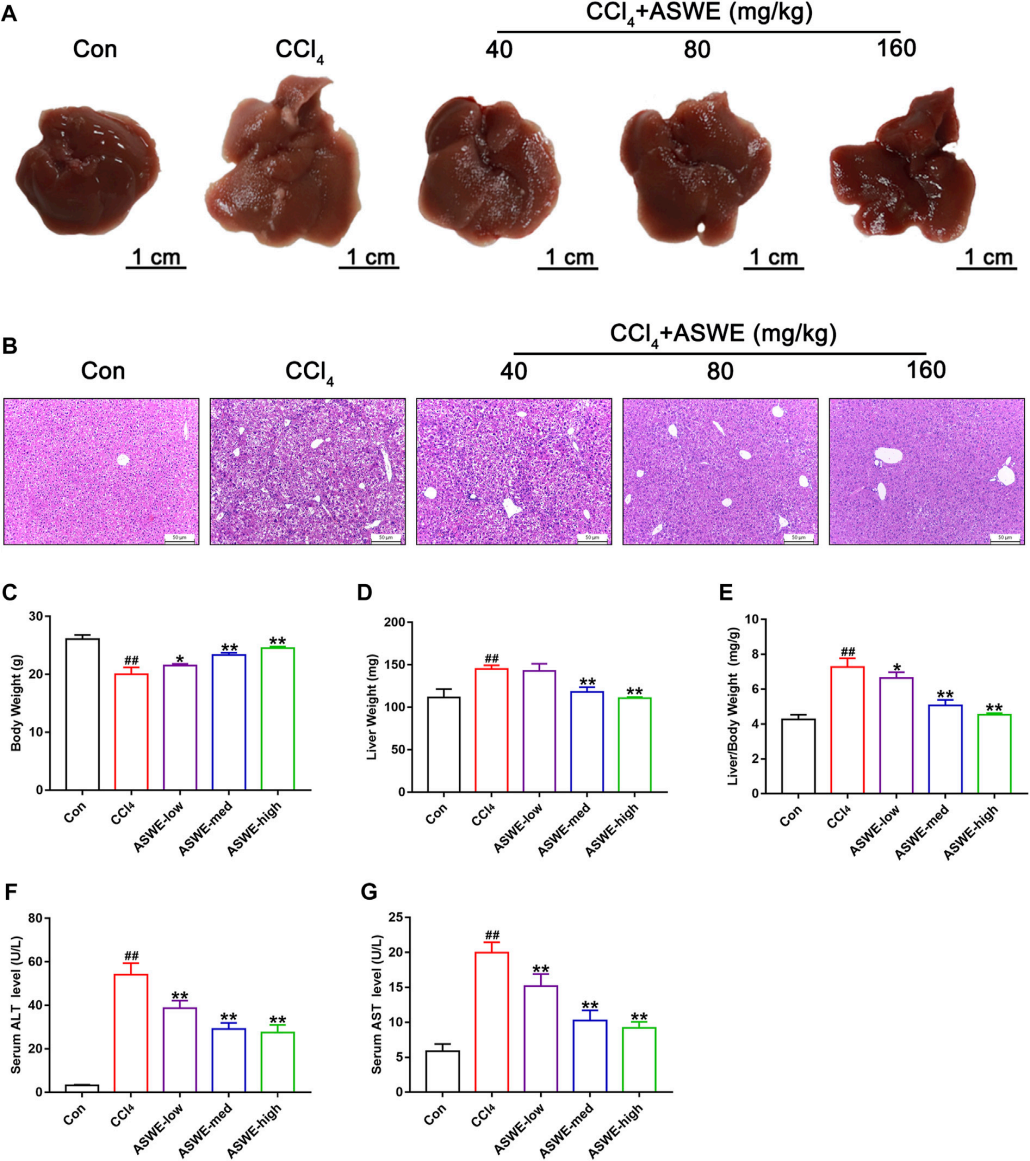
ASWE alleviated hepatic injury in CCl_4_-induced mice. **(A)** Representative liver photographs from control, model, and drug-treated groups (scale bar: 1 cm); **(B)** H&E staining (scale bar: 50 μm); **(C–E)** Body weight, liver weight and liver/body weight index (%) in mice; **(F,G)** The levels of ALT and AST in the serum. Data are presented as mean ± SD (*n* = 6). ^#^ *p* < 0.05, ^##^ *p* < 0.01, compared with control group; *p < 0.05, **p < 0.01, compared with CCl_4_ group.

To determine the effect of ASWE on the occurrence and development of hepatic injury and fibrosis in CCl_4_-induced mice, we firstly examined the morphological changes of liver tissues, as shown in Figure 2A, the livers of the control group were smooth, soft and dark red. However, the livers of the model group had a rough, lusterless surface with speckled and granular lesions and a hard texture, indicating that the liver was severely damaged. In contrast, after treatment with ASWE, the livers were smooth with relatively rosy color and soft texture, and the number of grain was obviously reduced. In addition, H&E staining of liver tissue samples from the model group revealed severe inflammatory infiltration and hepatocyte necrosis in portal areas. However, ASWE treatment significantly reduced the abnormal histological changes listed above (Figure 2B).

Similarly, CCl_4_ occasioned a considerable increase in liver weight and reduce in body weight, which led to an increase in liver index values. However, ASWE clearly dose-dependently ameliorated the symptoms listed above (Figures 2C–E). Moreover, to further explore the protective effects of ASWE on HF, we measured the transaminase activity including ALT and AST in the serum, which were the key hallmarks of liver function (Suciu et al., 2020). Compared to the control group, the serum levels of ALT and AST were significantly increased in the model group. Interestingly, the serum levels of ALT and AST were markedly dose-dependently reduced by ASWE treatment as compared with the model group (Figures 2F, G). Taken together, these results imply that ASWE treatment protects mice from liver damage induced by CCl_4_ treatment *in vivo*.

### 3.4 ASWE ameliorated collagen deposition and the expression of fibrotic markers in CCl_4_-induced HF mice

**FIGURE 3:**
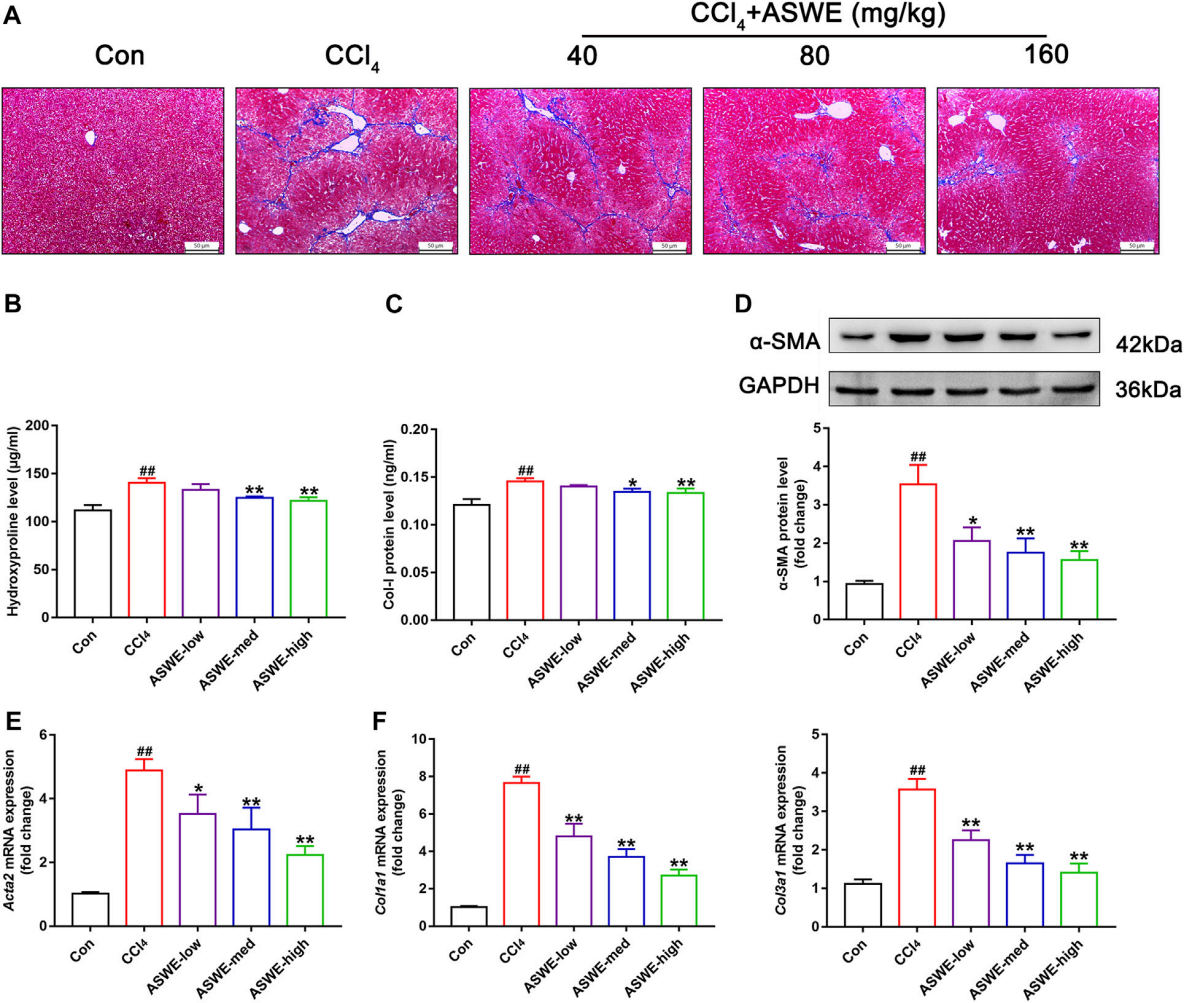
ASWE ameliorated collagen deposition and the expression of fibrotic markers in CCl_4_-induced HF mice. **(A)** Masson staining (scale bar: 50 μm); **(B,C)** The levels of Hyp and Col I in the serum (*n* = 6); **(D)** The protein expression of α-SMA (*n* = 3); **(E,F)** The mRNA expressions of fibrotic markers (*Acta2*, *Col1a1*, and *Col3a1*). Data are presented as mean ± SD (*n* = 4). ^#^ *p* < 0.05, ^##^ *p* < 0.01, compared with control group; *p < 0.05, **p < 0.01, compared with CCl_4_ group.

Considering that collagen deposition is one of the important features of HF (Zhang et al., 2022). We examined the degree of collagen deposition in CCl_4_-induced HF mice. Masson staining results showed severe collagen deposition in the CCl_4_ group. However, ASWE treatment clearly dose-dependently alleviated collagen deposition as compared with CCl_4_ treatment (Figure 3A). Meanwhile, the biomarkers of fibrogenesis, including Hyp and Col Ⅰ, were further examined by corresponding kits. As shown in Figures 3B, C, the levels of Hyp and Col Ⅰ in serum in the CCl_4_ group were significantly higher than that in the control group, and ASWE treatment significantly reduced the levels of Hyp and Col Ⅰ.

HF is always accompanied with activation of HSCs which are the primary source of activated myofibroblasts that produce ECM in the liver (Seki and Schwabe, 2015; Tsuchida and Friedman, 2017; Baghaei et al., 2022). Therefore, the expression levels of liver fibrosis marker α-SMA, which is closely related to activation of HSCs, was detected by Western blot and RT-qPCR. The results showed that the protein and mRNA expression of α-SMA was markedly enhanced in the CCl_4_ group. Whereas, ASWE treatment significantly inhibited the expression of this important fibrosis marker (Figures 3D, E). Moreover, the RT-qPCR results also showed that CCl_4_ treatment cause an excessive mRNA expression of *Col1a1* and *Col3a1*. And these abnormal expressions induced by CCl_4_ treatment were dose-dependently reversed by ASWE treatment (Figure 3F). Collectively, these results demonstrate that ASWE can inhibit collagen accumulation and the activation of HSCs in CCl_4_-induced HF mice, thereby alleviating liver fibrogenesis.

### 3.5 ASWE inhibited the activation of HSCs and suppressed inflammation *in vitro*

**FIGURE 4:**
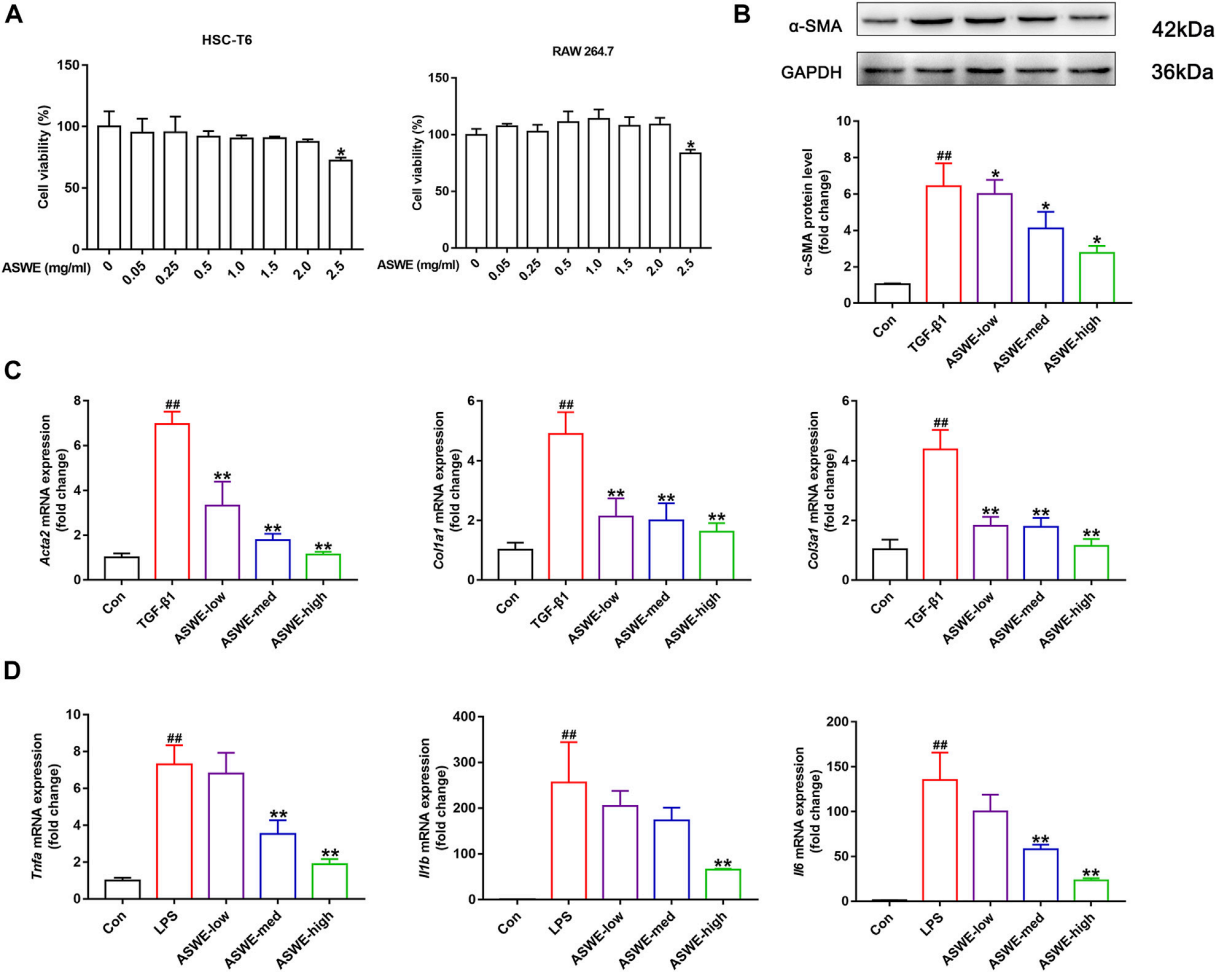
ASWE inhibited the activation of HSCs and suppressed Inflammation *in vitro*. **(A)** The viabilities of HSC-T6 and RAW264.7 cells; Data are presented as mean ± SD (*n* = 3). *p < 0.05, compared with control group; **(B)** The protein expression of α-SMA (*n* = 3); **(C)** The mRNA expressions of fibrotic markers (*Acta2*, *Col1a1*, and *Col3a1*). **(D)** The mRNA expressions of inflammatory factors (*Tnf-α*, *Il1β*, and *Il6*). Data are presented as mean ± SD (*n* = 4). ^#^ *p* < 0.05, ^##^ *p* < 0.01, compared with control group; *p < 0.05, **p < 0.01, compared with model group (TGF-β1 or LPS group).

To further investigate the antifibrotic effect of ASWE and its underlying mechanism, we used HSC-T6 cells and RAW264.7 cells for *in vitro* experiments. we first performed a CCK8 assay to assess whether ASWE had cellular toxicity on HSC-T6 cells and RAW264.7 cells. The results indicated that the viabilities of HSC-T6 and RAW264.7 cells were higher than 80% when the cells were treated with 0.05–2.0 mg/mL ASWE, while ASWE at 2.5 mg/mL exhibited significant inhibition to both cell types (Figure 4A). This results indicated that ASWE had no remarkable toxicity for HSC-T6 and RAW264.7 cells when the concentration of ASWE is lower than 2.0 mg/mL. This prompts us to select the appropriate doses of ASWE (0.25, 0.5, and 1 mg/mL) in the further experiments. After that, we further observed the expression of α-SMA protein in TGF-β-stimulated HSC-T6 cells. As shown in Figure 4B, the expression of α-SMA, which was promoted by the TGF-β1 stimulation, was inhibited by ASWE treatment in a dose-dependent manner. In addition, consistent with the *in vivo* results, ASWE downregulated the expressions of the genes that were related to collagen deposition in HSC-T6 cells, including *Acta2*, *Col1a1*, and *Col3a1* (Figure 4C). Inflammatory response is closely associated with HF, and the development of fibrosis usually leads to an increase in inflammatory factors (Parola and Pinzani, 2019). Therefore, the mRNA expression of *Tnf-α*, *Il1β*, and *Il6* was investigated by RT-qPCR analysis and the results showed that their expression was significantly augmented by the LPS stimulus and observably diminished by ASWE treatment in a dose-dependent manner in LPS-stimulated RAW 264.7 cells, and the high dose group (ASWE-high group) had the best effect. (Figure 4D). Overall, these results provide evidence that ASWE can inhibit HSCs activation and attenuate the inflammatory response.

### 3.6 Stat3 signaling pathway could be involved in the regulation of the anti-fibrotic process of ASWE

**FIGURE 5:**
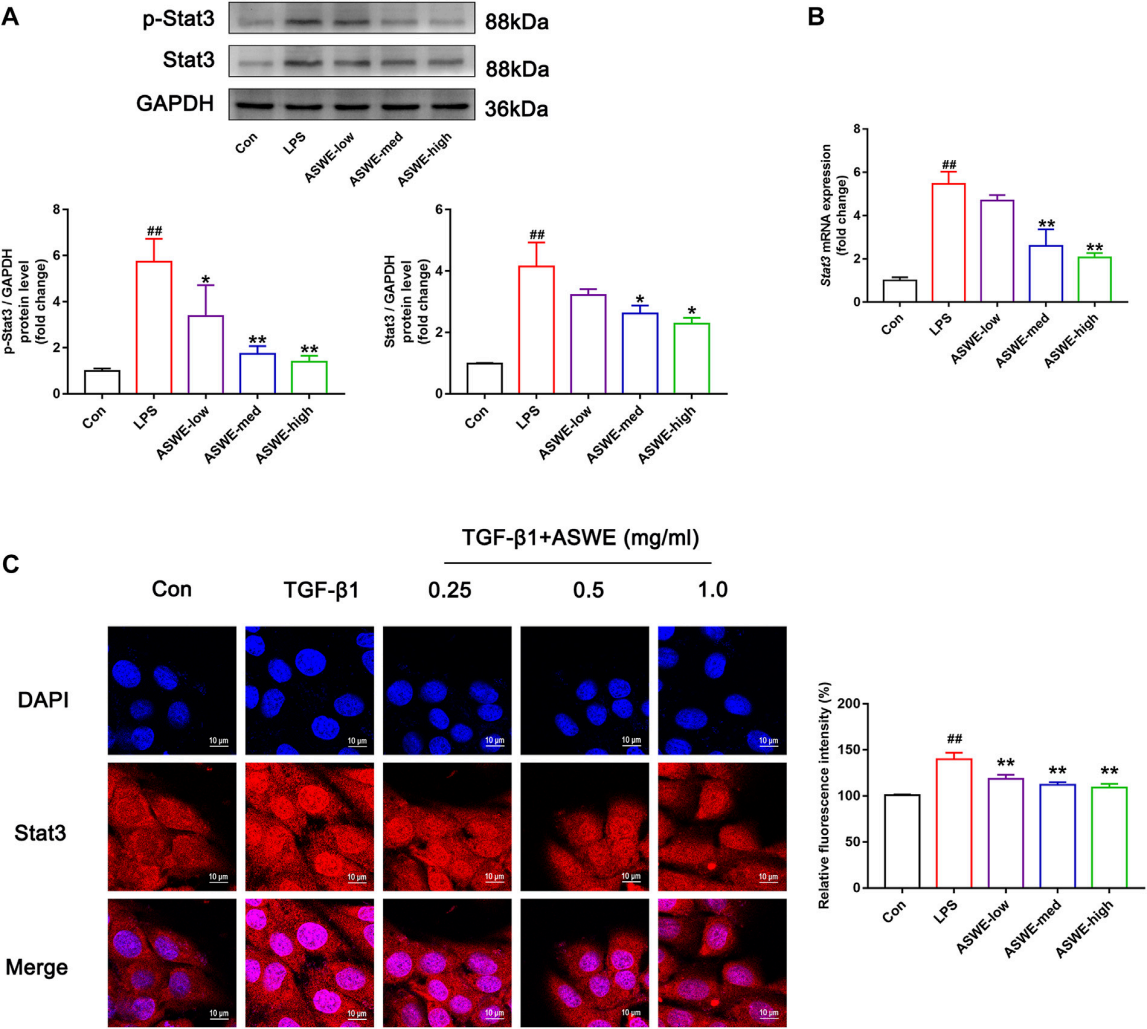
Stat3 signaling pathway could be involved in the regulation of the anti-fibrotic process of ASWE. **(A)** The protein expressions of p-Stat3 and total Stat3 (*n* = 3); **(B)** The mRNA expression of Stat3. Data are presented as mean ± SD (*n* = 4). ^#^ *p* < 0.05, ^##^ *p* < 0.01, compared with control group; *p < 0.05, **p < 0.01, compared with LPS group. **(C)** The subcellular location of STAT3 in HSC-T6 cells by immunofluorescent staining (scale bar: 10 μm).

In the above experiments, we found that ASWE had anti-inflammatory effects. Currently, domestic and international studies have confirmed that the Stat3 signaling pathway is closely linked to the secretion of pro-inflammatory factors and this pathway also plays a crucial role in the fibrosis process of liver (Guan et al., 2021) (Ma et al., 2022). Therefore, we speculated that ASWE is likely to have a regulatory role on the transcriptional activity of Stat3. To investigate the effect of ASWE on Stat3 signaling pathway, we examined the expression of Stat3 and the phosphorylation level of Stat3 at tyrosine 705 (p-Stat3), which is responsible for the activation of Stat3 (Wen et al., 1995) as well as the mRNA expression of *Stat3* gene. Apparently, the expressions of p-Stat3 and total Stat3 were both elevated and the expression of *Stat3* gene was upregulated in LPS-stimulated RAW 264.7 cells. Notably, ASWE treatment reversed LPS-induced upregulation of phosphorylated Stat3, total Stat3, and *Stat3* gene (Figures 5A, B). These observations suggest that ASWE treatment can reverse LPS-induced activation of Stat3.

After activation, Stat3 transported from the cytoplasm to the nucleus to regulate the transcription of its target genes (Kurdi and Booz, 2010). Thus, the nuclear translocation of Stat3 was investigated by detecting the subcellular distribution of Stat3 under confocal microscope. As shown in Figure 5C, the immunofluorescence staining results showed that Stat3 fluorescence was assembled in the nucleus of the TGF-β1-treated cells, whereas it was retained in the cytoplasm in the control cells. Administration of different doses of ASWE inhibited the nuclear shuttling of Stat3. Summarily, these results demonstrated that ASWE had an inhibitory effect on Stat3 activation and nuclear translocation. This suggests that ASWE is likely to alleviate the inflammatory response by inhibiting the activity of Stat3 signaling pathway, and thus exhibiting the antifibrotic effect.

### 3.7 ASWE restrained HSCs activation through suppression of the Stat3 signaling pathway

**FIGURE 6:**
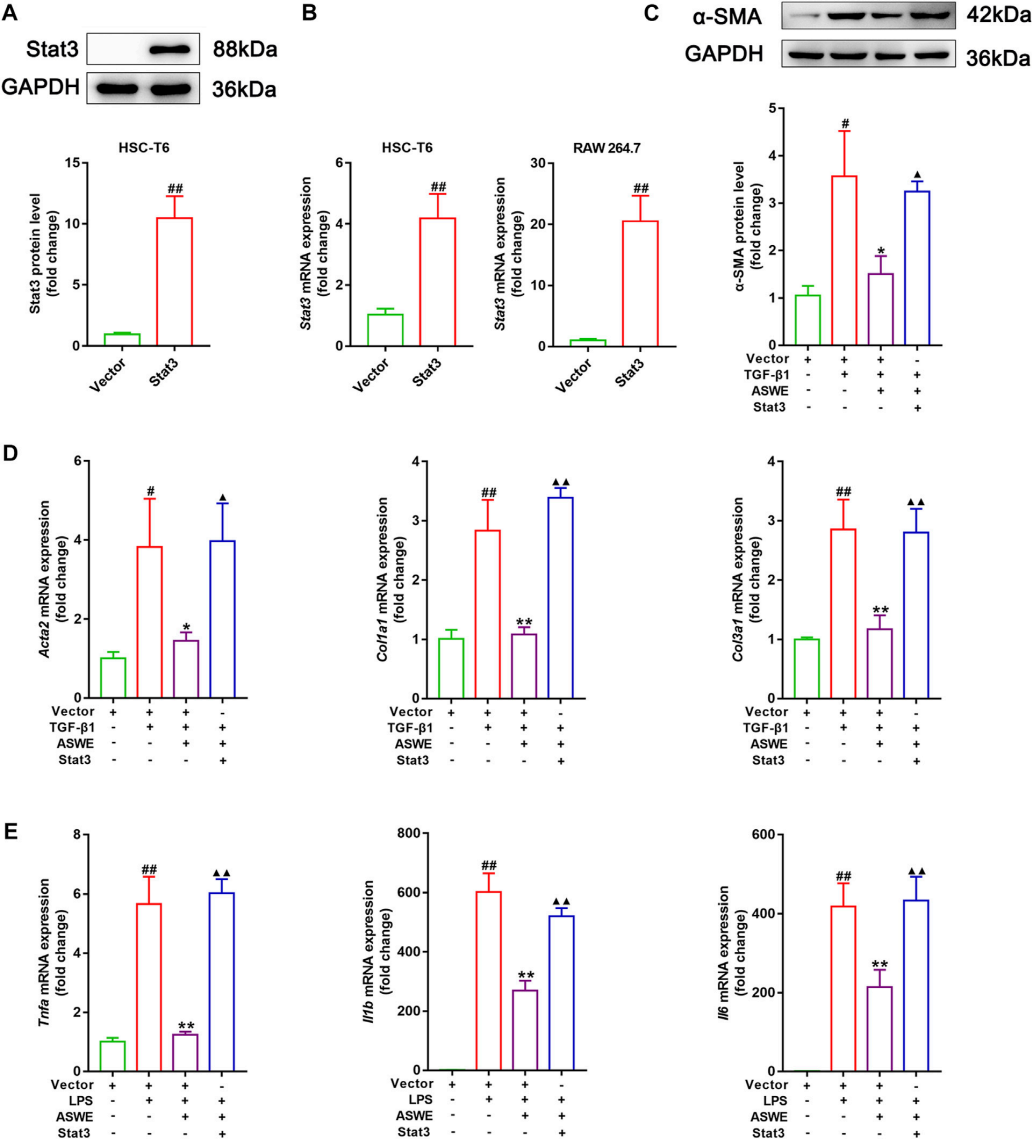
ASWE restrained HSCs activation through suppression of the Stat3 signaling pathway. **(A)** The protein expression of Stat3; **(B)** The mRNA expression of *Stat3*. **(C)** The protein expression of α-SMA (*n* = 3); **(D,E)** The mRNA expressions of *Acta2*, *Col1a1*, *Col3a1*, *Tnf-α*, *Il1β*, and *Il6*. Data are expressed as mean ± SD (*n* = 4). ^#^ *p* < 0.05 and ^##^ *p* < 0.01 vs. control group; ^∗^ *p* < 0.05 and ^∗∗^ *p* < 0.01 vs. model group (TGF-β1 or LPS group); ^▲^ *p* < 0.05 and ^▲▲^ *p* < 0.01 vs. AHWE group.

The aforementioned experiments results prompted us to further clarify the significance of the Stat3 signaling pathway in inhibiting fibrosis and inflammation of ASWE. Therefore, we used the Stat3 plasmid to overexpress Stat3 for the next experiments. As shown in Figures 6A, B, Stat3 was successfully overexpressed in HSC-T6 cells and RAW 264.7 cells. Furthermore, as shown in Figure 6C, ASWE inhibited the elevated expression of α-SMA protein induced by TGF-β1 in HSC-T6 cells, but this effect was reversed by Stat3 overexpression. Meanwhile, Stat3 overexpression enhanced the mRNA expression levels of the fibrosis markers *Acta2*, *Col1a1*, and *Col3a1* and the inflammation markers *Tnf-α*, *Il1β* and *Il6*, which weakened the inhibitory effect of ASWE (Figures 6D, E). These results provide evidence that ASWE inhibits the activation of HSCs by suppressing the Stat3 signaling pathway.

### 3.8 ASWE inhibited the activation of Stat3 in CCl4-induced HF mice

**FIGURE 7:**
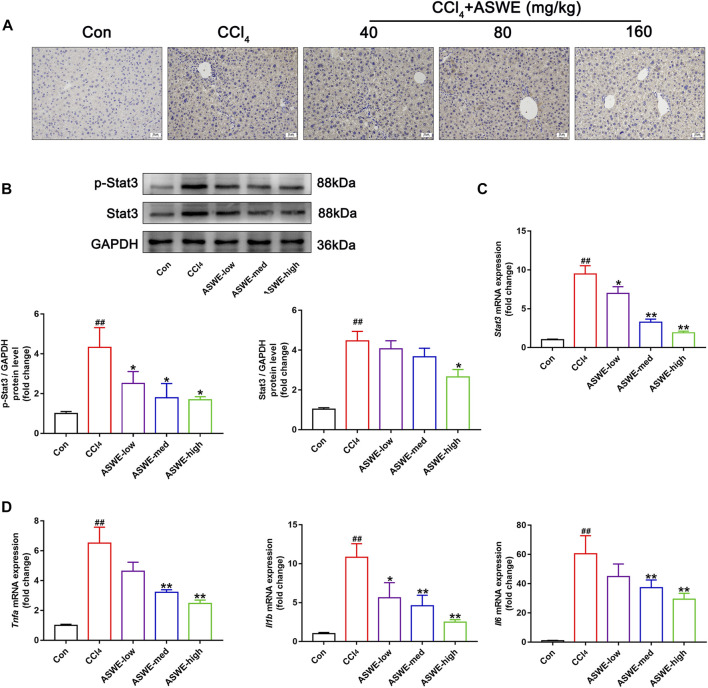
ASWE inhibited the activation of Stat3 pathway in CCl_4_-induced HF mice. **(A)** The protein expression of Stat3 in the livers of C57BL/6J mice was detected by immunohistochemistry (scale bar: 10 µm). **(B)** The protein expressions of p-Stat3 and total Stat3 in mice (*n* = 3); **(C)** The mRNA expression of Stat3 in mice. **(D)** The mRNA expressions of inflammatory factors (*Tnf-α*, *Il1β*, and *Il6*) in mice. Data are presented as mean ± SD (*n* = 4). ^#^ *p* < 0.05, ^##^ *p* < 0.01, compared with control group; *p < 0.05, **p < 0.01, compared with CCl_4_ group.

In order to verify that ASWE indeed exerts an antifibrotic effect by inhibiting the activation of HSCs through suppression of the Stat3 signaling pathway. Immunohistochemistry assay was performed to observe the expression of Stat3 in CCl_4_-induced HF mice. As the results showed, significantly higher Stat3 expression in mouse livers with CCl_4_ treatment was found as compared with the control group (Figure 7A). Thus, it can be seen that CCl_4_ significantly promoted the expression of Stat3, while ASWE treatment reduced the expression of Stat3 in a dose-dependent manner. Thereafter, we further observed the protein expression of p-Stat3 and Stat3 in liver tissues by Western blot. Meanwhile, we also performed RT-qPCR analysis to examine the expression level of *Stat3* gene. The results showed that ASWE significantly reduced the elevation of the phosphorylation level of Stat3 at tyrosine 705 (p-Stat3), the increase of Stat3 protein expression and the upregulation of *Stat3* gene induced by CCl_4_ stimulation in a dose-dependent manner (Figures 7B, C). The anti-inflammatory effect of ASWE in the livers of HF mice was also confirmed by RT-qPCR analysis. As shown in Figure 7D, ASWE inhibited the expression of inflammation-related genes, including *Tnf-α*, *Il1β*, and *Il6*, which was elevated by CCl_4_ treatment. The above *in vitro* and *in vivo* results consistently demonstrated that ASWE could inhibit the activation of HSCs by inhibiting the Stat3 signaling pathway, and ultimately alleviate HF.

## 4 Discussion

HF is a reversible wound-healing response during liver injury that is characterized by excessive deposition of ECM. Long-term persistent development of HF will lead to cirrhosis and even liver cancer, threatening public health. Currently, clinically effective and safe drugs and treatments for HF are lacking. Therefore, exploring and developing new drugs and therapies to treat HF is a research challenge (Bataller and Brenner, 2005; Tsochatzis et al., 2014). Traditional Chinese medicine (TCM) has unique advantages and development prospects in the treatment of chronic inflammation-related diseases such as HF (Cheng et al., 2017; Liu et al., 2021b; Ran et al., 2021). TCM has been used for centuries as a complementary and alternative treatment to prevent liver fibrosis. At present, some compounds in TCM inhibit liver fibrosis, such as paeoniflorin (Chen et al., 2012), salvianolic acid B (Li et al., 2012), quercetin (Wu et al., 2011), puerarin (Li et al., 2013), tetrandrine (Ezhilarasan et al., 2012), matrine (Zhang et al., 2001), silybin (Zhang et al., 2001), and oxymatrine (Chai et al., 2012). *Amydrium sinense* (Engl.) H. Li is a unique herbal that is used to treat a variety of common diseases. In the current study, we demonstrated the potential antifibrotic effect of the water extract of *Amydrium sinense* (Engl.) H. Li in a CCl_4_-induced chronic liver fibrosis mouse model for the first time. Mechanistically, we revealed that ASWE protected the liver by inhibiting HSC activation by suppressing Stat3 signaling pathway.

First, the chemical components of ASWE were analysed by a Q-Orbitrap high-resolution mass spectrometer (Figure 1; Table 2) and the main targets of compounds in ASWE were predicted using the, ETCM database. A total of 250 putative targets of ASWE were predicted and 998 HF-related genes were collected. Screening showed that of the 250 ASWE targets, 44 were also known therapeutic targets for treating HF. To reflect the relationship between targets and compounds, we have used Cytoscape 3.7.1 to map out the compound-target relation network diagram, the compound-target diagram consisted of 52 nodes (8 core active compound nodes and 44 active target nodes). Then, to identify the biological function of the core targets, GO and KEGG enrichment analyses of the 44 core targets were performed using the DAVID online tool, 8 vital biological processes were obtained by mapping targets. The majority of these targets were closely related to intracellular receptor signaling pathway, cellular response to external stimulus, regulation of lipid metabolic process, regulation of inflammatory response and fatty acid metabolic process. These biological processes are partially associated with the mechanism of HF. Cellular component analysis revealed that these targets are distributed in the transcription regulator complex, extrinsic component of cytoplasmic side of plasma membrane, cytoplasmic side of plasma membrane, extrinsic component of plasma membrane, cytoplasmic side of membrane. Molecular function analysis showed that these targets are related to nuclear receptor activity, ligand-activated transcription factor activity, RNA polymerase Il-specific DNA-binding transcription factor binding and DNA-binding transcription factor binding. These analytical results suggested that inflammatory response might be involved in the anti-antifibrotic process of ASWE against HF. The biological functions and pathways involving 44 targets in the treatment of HF were evaluated via KEGG enrichment analysis. Overall, the targets were notably related to the non-alcoholic fatty liver disease, alcoholic liver disease, hepatitis C, hepatitis B (Supplementary Figure S1). These results strongly suggested that ASWE might play an important role in protecting liver.

Intraperitoneal injection of CCl_4_ has been widely used to establish a stable fibrotic model that partially resembles liver fibrosis in humans. CCl_4_ is metabolized by cytochrome P450 enzyme (CYP2E1) to generate free radicals in the liver, destroying the integrity of the cell membrane and resulting in lipid peroxidation and hepatic injury (Slater et al., 1985; El-Agroudy et al., 2016). Repeated exposure to CCl_4_ eventually enhances liver fibrogenesis (Tsukamoto et al., 1990). In the present study, we established a mouse model of CCl_4_-induced hepatic fibrosis and evaluated the protective effect of ASWE against fibrosis. We used three different doses (40 mg/kg, 80 mg/k and 160 mg/kg) of ASWE to assess its effects. The results showed that after continuous intraperitoneal injection of the CCl_4_-olive oil solution for 4 weeks, the mice in the HF model group showed deterioration of liver morphology, increased liver volume, less weight gain, and an elevated liver index, indicating severe liver injury, which was consistent with the clinical manifestations of HF (Ekser et al., 2012). In contrast, treatment with ASWE ameliorated these changes. In addition, H&E staining showed obvious pathological changes in the liver tissue of HF mice, such as inflammatory cell infiltration, massive adipose tissue vacuolization and hepatocyte necrosis. However, ASWE significantly attenuated these abnormal changes in liver fibrosis (Figures 2A–E). ALT and AST are two validated liver enzymes that are commonly used to assess liver function. Elevated serum levels of ALT and AST often reflect the degree of hepatocyte injury (Giannini et al., 2005). The results showed that ASWE treatment abrogated the increased levels of ALT and AST in the model group (Figures 2F, G), which provided conclusive evidence that ASWE protected hepatocytes from chronic injury.

In the normal liver, HSCs are in a quiescent and nonproliferating state, and HSC activation is crucial in liver fibrogenesis. During liver fibrosis, α-SMA is typically known as a biomarker of HSC activation and fibrogenesis and represents the primary pathophysiological event (Friedman, 2008). Activated HSCs play a crucial role in the excessive synthesis and deposition of ECM by secreting collagen and insoluble fibrin, which is one of the main components of the ECM (Higashi et al., 2017). Evidence has indicated that the excessive deposition of ECM can be reduced by inhibiting the activation of HSCs (Tsochatzis et al., 2014; Magdaleno et al., 2018). Therefore, inhibiting HSC activation has been recognized as an effective strategy for the prevention and treatment of liver fibrosis (Tomita et al., 2014; Pawlak et al., 2015). In this study, the Masson staining results showed that a large number of collagen fibers were formed in the liver tissue of the model group. However, different doses of ASWE reduced the accumulation of collagen fibers. Furthermore, we measured serum levels of Col-I and HYP, which are characteristic of collagen fibers and reflect the degree of liver fibrosis (Lee et al., 2005), and showed that ASWE could reduce collagen accumulation in mouse livers induced by CCl_4_ stimulation. The Western blot results showed that the high expression of α-SMA protein in the model group was reduced by ASWE treatment. The RT-qPCR results further showed that ASWE could reverse the high expression of fibrosis-related genes in the model group, including *Acta2*, *Col3a1*, and *Col1a1* (Figure 3). These results suggested that ASWE could inhibit the activation of HSCs and the excessive deposition of ECM induced by CCl_4_, thereby suppressing HF, suggesting that ASWE was an effective and potent agent for treating liver fibrosis.

Moreover, we established an *in vitro* model of HSC activation by stimulating HSC-T6 cells with TGF-β1, a crucial profibrogenic factor. In accordance with the *in vivo* results, ASWE significantly reduced the elevated protein expression of α-SMA in TGF-β1-treated HSC-T6 cells. In addition, ASWE reversed the high expression of fibrosis-related genes (*Acta2, Col3a1, Col1a1*) induced by TGF-β1, suggesting that ASWE could inhibit HSC activation and promote ECM degradation. Inflammation leads to the activation of effector cells, which causes the deposition of ECM. Cytokines released from inflammatory cells play a key role in the underlying pathogenesis of liver fibrosis (Lieber, 2004). Proinflammatory cytokines, including Tnf-α, Il1β, and Il6, are released from innate immune cells and promote fibrogenesis by active HSCs (Wu et al., 2020). Thus, we established an *in vitro* model of cellular inflammation by stimulating RAW 264.7 cells with LPS. As the results showed, ASWE significantly abrogated the increased expression of inflammation-related genes (*Tnf-α, Il1β*, *and Il6*) induced by LPS stimulation (Figure 4). Therefore, we showed that ASWE could inhibit HSC activation and the inflammatory response *in vitro*. Thus, we speculate that ASWE may protect against liver fibrosis injury through anti-inflammatory effects.

It has been well documented that JAK2/Stat3 signaling is constantly activated during the progression of HSC activation, leading to various pathological manifestations of HF (Zhao et al., 2021). Blockade of the Stat3 signaling pathway impairs the morphological transdifferentiation of HSCs and reduces the expression of profibrotic genes (Wang et al., 2018; Choi et al., 2019). In unstimulated cells, Stat3 is inactive and located in the cytoplasm. The binding of Stat3-related cytokines to their receptors activates the receptor-associated Janus tyrosine kinases (JAK), which phosphorylates Stat3. Stat3 dimerizes in response to tyrosine phosphorylation at site 705, which results in its nuclear translocation, and Stat3 functions as a transcriptional factors for downstream genes, such as inflammation-related genes (*Tnf-α, Il1β*, *and Il6*). Inhibition of Stat3 phosphorylation could inhibit Stat3 nuclear localization (Xiang et al., 2018). We hypothesized that the Stat3 signaling pathway might be involved in the regulation of the antifibrotic effect of ASWE. In subsequent studies, we further found that the antifibrotic activity of ASWE was closely related to the Stat3 signaling pathway *in vitro* and *in vivo*. ASWE could inhibit the phosphorylation and activation of Stat3 and inhibit its nuclear translocation (Figure 5). In addition, the overexpression of Stat3 elevated the expression levels of α-SMA protein and *Acta2*, *Col1a1*, *Col3a1*, *Tnf-α*, *Il6*, and *Il1β* mRNA (Figure 6). These results indicated that ASWE could weaken the effects of ASWE on suppressing HSC activation and attenuating inflammation, and ultimately aggravate the progression of HF.

Based on these *in vitro* experiments, we confirmed that ASWE exerted its antifibrotic effects by mediating the Stat3 signaling pathway to inhibit the activation of HSCs. Notably, we verified the consistent regulatory mechanism in CCl_4_-induced mice. ASWE inhibited the expression and distribution of Stat3 in liver tissues, and decreased the expression levels of p-Stat3 and Stat3 protein and *Stat3* mRNA induced by CCl_4_. CCl_4_ upregulated inflammatory genes such as *Tnf-α*, *Il6*, and *Il1β* (Figure 7), leading to the formation of a hepatic inflammatory microenvironment and promoting HF. In contrast, ASWE significantly reversed these detrimental changes. These results provided evidence that the regulation of the Stat3 signaling pathway was involved in the *in vivo* suppression of HF by ASWE treatment, which was consistent with the *in vitro* findings.

## 5 Conclusion

**FIGURE 8:**
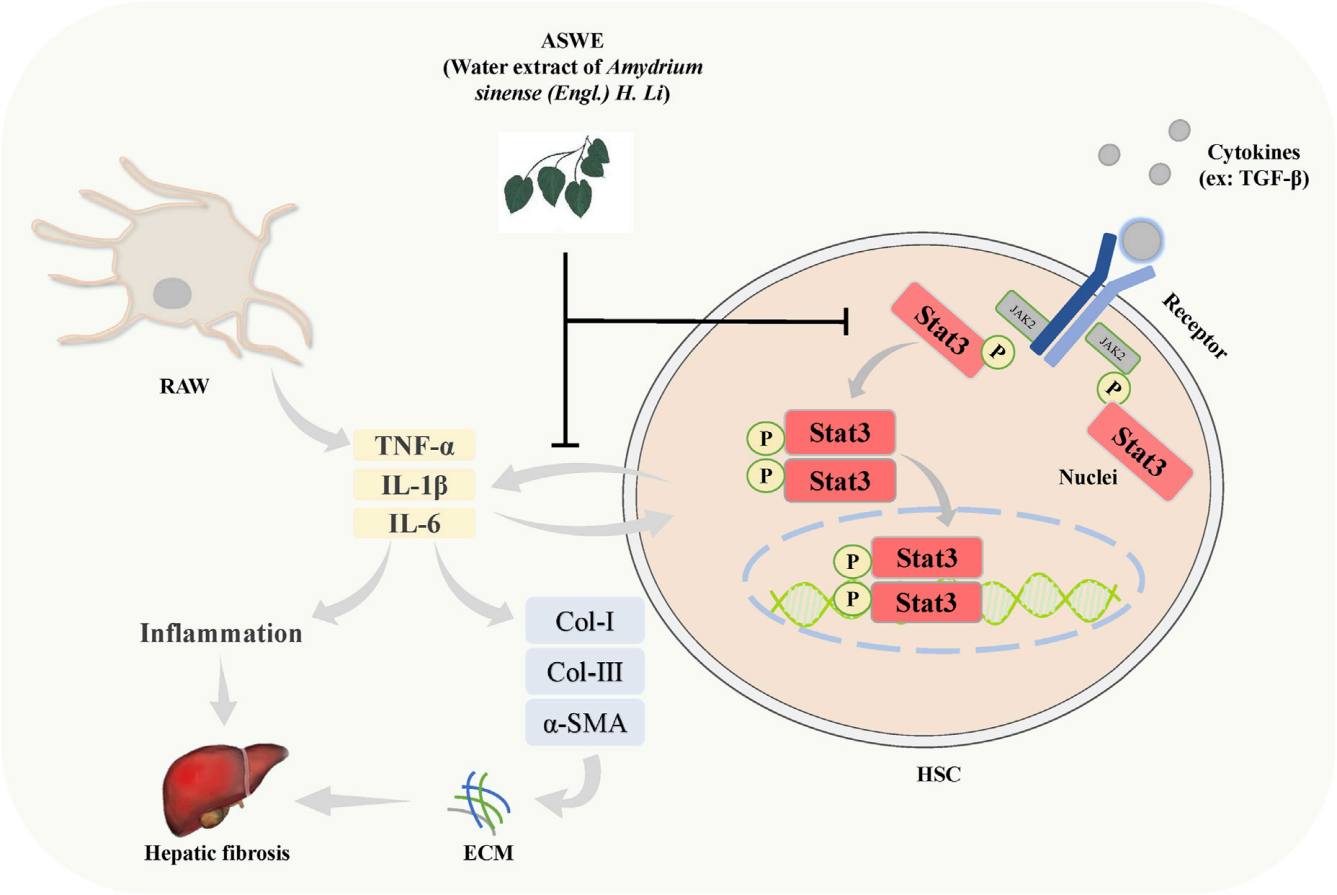
Schematic diagram of the protective role of ASWE in HF via suppressing hepatic stellate cell activation through inhibiting Stat3 signaling. AHWE inhibited the expression and distribution of Stat3 in liver tissues, and decreased the expression levels of p-Stat3 and Stat3 protein and *Stat3* mRNA induced by CCl_4_. CCl_4_ upregulated the inflammatory genes such as *Tnf-α*, *Il6*, and *Il1β*, leading to the formation of a hepatic inflammatory microenvironment and promoting HF. In contrast, ASWE significantly reversed these detrimental changes.

The present study demonstrated that ASWE effectively improved CCl_4_-induced hepatic fibrosis induced in mice. The underlying molecular mechanism may involve ASWE-mediated inhibition of HSC activation and the inflammatory response by suppressing the Stat3 signaling pathway (Figure 8). Therefore, our data provide further support for the antifibrotic mechanism of ASWE. ASWE is expected to become an effective drug for the treatment of liver fibrosis and provide an effective reference and new ideas for clinical treatments.

## Data Availability

The original contributions presented in the study are included in the article/Supplementary Material, further inquiries can be directed to the corresponding authors.

## Supplementary material

SUPPLEMENTARY FIGURE S1 Network of compound and disease targets and enrichment of GO and KEGG analysis. **(A)** The common targets of compound targets and HF targets. **(B)** Compound-HF target network. **(C)** The GO biological process (BP), cellular component (CC) and molecular function (MF) analysis diagram. **(D)** Enriched KEGG pathways of potential key targets.

## Abbreviations

Acta2, Actin alpha 2; AS, *Amydrium sinense (Engl.) H. Li*; ASWE, water extract of *Amydrium sinense (Engl.) H. Li*; ALT, Alanine transaminase; AST, Aspartate transaminase; CCl_4_, carbon tetrachloride; Col Ⅰ, Collagen 1; Col Ⅲ, Collagen 3; Col1a1, Collagen 1a1; Col3a1, Collagen 3a1; ECM, extracellular matrix; GAPDH, Glyceraldehyde-3-phosphate dehydrogenase; H&E, Hematoxylin and eosin; HF, Hepatic fibrosis; HSCs, Hepatic stellate cells; LPS, lipopolysaccharide; Il1β, Interleukin-1 beta; Il6, Interleukin-6; Stat3, Signal transducers and activators of transcription 3; Tnf-α, Tumor necrosis factor-α; TGF-β, Transforming growth factor beta; α-SMA, alpha smooth muscle actin.
