# Supplementary figures and images for: Aqueous extract of Amydrium sinense (Engl.) H. Li alleviates hepatic fibrosis by suppressing hepatic stellate cell activation through inhibiting Stat3 signaling
Source: Front Pharmacol. 2023 Jun 13;14:1101703. doi: 10.3389/fphar.2023.1101703 (PMC10293641; doi:10.3389/fphar.2023.1101703)

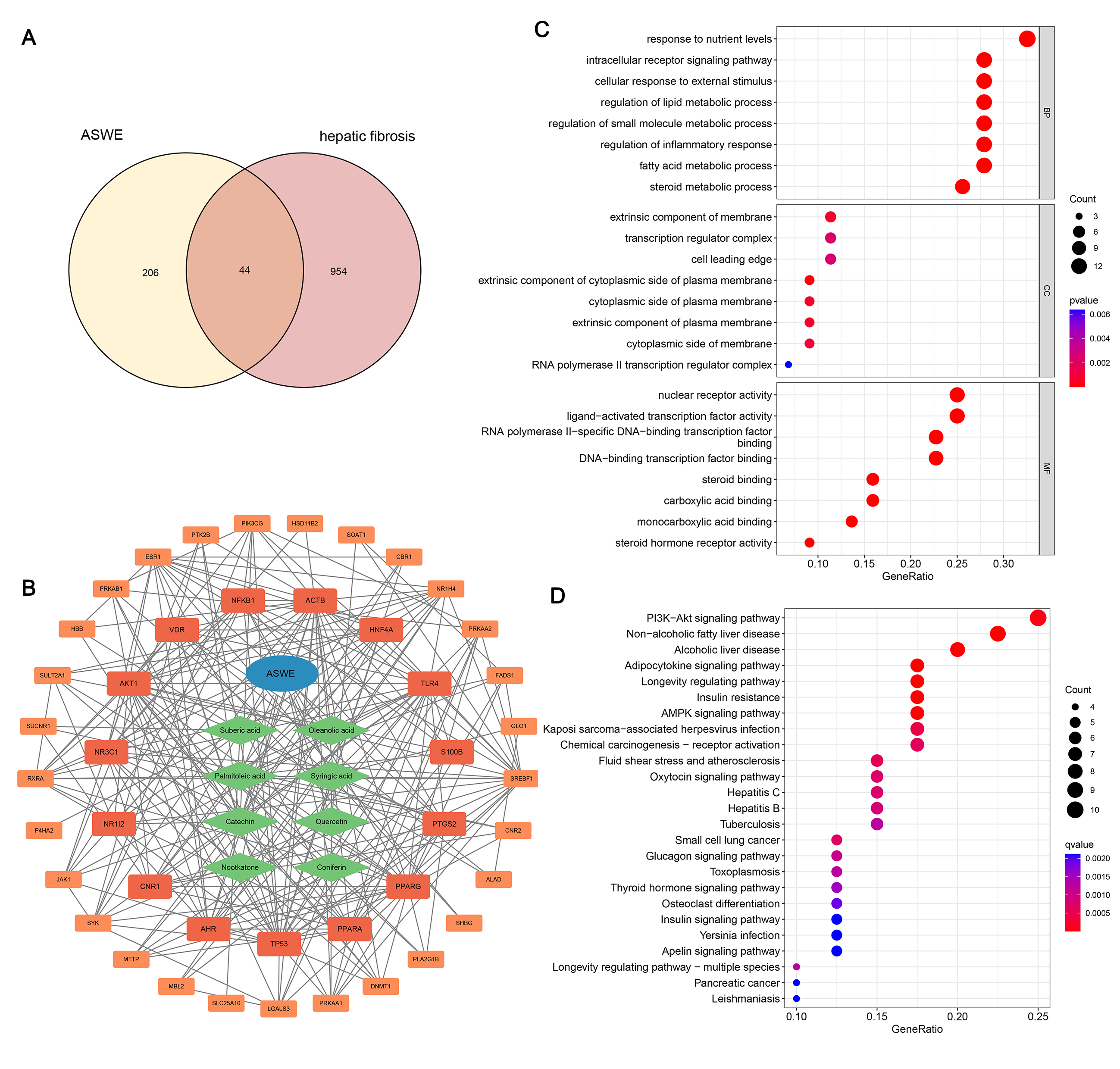

Supplement: Supplementary file 1 [file Image1.TIF]
